# Inulin-type fructan degradation capacity of interesting butyrate-producing colon bacteria and cross-feeding interactions of *Faecalibacterium prausnitzii* DSM 17677^T^ with bifidobacteria

**DOI:** 10.1186/2049-3258-72-S1-O6

**Published:** 2014-06-06

**Authors:** Frédéric Moens, Audrey Rivière, Marija Selak, Luc De Vuyst

**Affiliations:** 1 Research Group of Industrial Microbiology and Food Biotechnology (IMDO), Faculty of Sciences and Bio-engineering Sciences, Vrije Universiteit Brussel, Pleinlaan 2, B-1050 Brussels, Belgium

## Background

Inulin-type fructans have already been studied with respect to their stimulation of bifidobacteria and butyrate-producing colon bacteria, such as *Anaerostipes caccae* and *Roseburia* spp. However, much less is known about their effects on other butyrate-producing colon bacteria, such as *Butyricicoccus pullicaecorum*, *Eubacterium* spp. and *Faecalibacterium prausnitzii* and the interactions of these species with bifidobacteria. This study aimed at investigating the kinetics of inulin-type fructan degradation and organic acid and gas production by *B. pullicaecorum* DSM 23266^T^, *E. hallii* L2-7, *E. rectale* CIP 105953^T^, and *F. prausnitzii* DSM 17677^T^ and the possible interactions between *F. prausnitzii* DSM 17677^T^ and bifidobacteria.

## Materials and methods

All butyrate-producing strains were studied during screening experiments (100-ml scale) and monoculture fermentations (1.5-l scale) in a medium for colon bacteria (MCB) containing either fructose, oligofructose, or long-chain inulin as an energy source, supplemented with acetate. Coculture fermentation experiments (1.5-l scale) in MCB were performed with *F. prausnitzii* DSM 17677^T^ and *Bifidobacterium breve* Yakult, *Bifidobacterium adolescentis* LMG 10734, *Bifidobacterium angulatum* LMG 11039^T^ (oligofructose), and *Bifidobacterium longum* LMG 11047 (oligofructose or inulin as a substrate).

## Results

*Butyricicoccus pullicaecorum* DSM 23266^T^ and *E. hallii* L2-7 degraded fructose only, resulting in the production of butyrate, H_2_ and CO_2_. *Eubacterium rectale* CIP 105953^T^ produced lactate and butyrate as well as H_2_ and CO_2_ out of fructose and inulin-type fructans. *Faecalibacterium prausnitzii* DSM 17677^T^ produced butyrate, formate, and traces of lactate, together with CO_2_ out of fructose, oligofructose, and inulin. Both oligofructose-consuming, butyrate-producing strains degraded all oligofructose fractions simultaneously, indicating an extracellular degradation mechanism. During coculture fermentation experiments, oligofructose (by all bifidobacteria, except for *B. breve* Yakult) and inulin (by *B. longum* LMG 11047) were converted into acetate, lactate, and formate. *Faecalibacterium prausnitzii* DSM 17677^T^ was cross-fed on this acetate resulting in the production of its metabolites. However, only low amounts of butyrate were produced during the coculture fermentations with *B. angulatum* LMG 11039^T^ and *B. longum* LMG 11047, since *F. prausnitzii* DSM 17677^T^ did not manage to compete well for the oligofructose substrate in the presence of *B. angulatum* LMG 11039^T^ and *B. longum* LMG 11047, and for the inulin substrate in the presence of *B. longum* LMG 11047.

## Conclusion

Besides cross-feeding interactions between bifidobacteria and butyrate-producing colon bacteria, competition for the available inulin-type fructans between these colon bacteria may occur. As a result, fast degraders such as bifidobacteria are favoured compared to acetate-depending butyrate producers.

